# Bulbospinal nociceptive ON and OFF cells related neural circuits and transmitters

**DOI:** 10.3389/fphar.2023.1159753

**Published:** 2023-04-20

**Authors:** Bingxue Peng, Yingfu Jiao, Yunchun Zhang, Shian Li, Sihan Chen, Saihong Xu, Po Gao, Yinghui Fan, Weifeng Yu

**Affiliations:** ^1^ Department of Anesthesiology, Renji Hospital, Shanghai Jiao Tong University School of Medicine, Shanghai, China; ^2^ Key Laboratory of Anesthesiology (Shanghai Jiao Tong University), Ministry of Education, Shanghai, China

**Keywords:** rostral ventromedial medulla, pain, neural circuit, transmitter, ON cell, OFF cell

## Abstract

The rostral ventromedial medulla (RVM) is a bulbospinal nuclei in the descending pain modulation system, and directly affects spinal nociceptive transmission through pronociceptive ON cells and antinociceptive OFF cells in this area. The functional status of ON and OFF neurons play a pivotal role in pain chronification. As distinct pain modulative information converges in the RVM and affects ON and OFF cell excitability, neural circuits and transmitters correlated to RVM need to be defined for an in-depth understanding of central-mediated pain sensitivity. In this review, neural circuits including the role of the periaqueductal gray, locus coeruleus, parabrachial complex, hypothalamus, amygdala input to the RVM, and RVM output to the spinal dorsal horn are discussed. Meanwhile, the role of neurotransmitters is concluded, including serotonin, opioids, amino acids, cannabinoids, TRPV1, substance P and cholecystokinin, and their dynamic impact on both ON and OFF cell activities in modulating pain transmission. Via clarifying potential specific receptors of ON and OFF cells, more targeted therapies can be raised to generate pain relief for patients who suffer from chronic pain.

## 1 Introduction

Central sensitization plays a significant role in the endogenous modulation of nociceptive transmission (Woolf, 1983). The descending pain modulation system influences the sensitization of central nociceptive transmission predominantly by altering the interconnections with the rostral ventromedial medulla (RVM) (Francois et al., 2017). Therefore, it is essential to define a role for RVM in pain modulation.

Located in the lower bulbospinal area, the RVM mainly consists of the nucleus raphe magnus (NRM), the nucleus reticularis gigantocellularis-pars alpha, and the nucleus paragiganto-cellularis lateralis (Zhuo and Gebhart, 1990). Regarding its function, it has been reported that when experiencing noxious heat stimuli or continuous neuropathic pain, not only the contralateral RVM but also the ipsilateral/median RVM activity is increased, as indicated in a functional magnetic resonance imaging (fMRI) study of human supraspinal structures (Rempe et al., 2015; Mills et al., 2020). Furthermore, in opioid-mediated, attentional, and placebo analgesia, a significant increase in correlated voxels was observed primarily in the middle and ventral aspects of the RVM (Crawford et al., 2021; Oliva et al., 2022). In addition, other investigations have subsequently conveyed the importance of the RVM in modulating descending pain in male and female animal models (Drake et al., 2007; M. M. Heinricher et al., 2009). These findings confirmed the distinctive role of the RVM as the main gatekeeper of descending pain modulation and facilitating bidirectional or inhibitory pain modulatory effects. Moreover, variations in RVM neuronal responses to nociceptive stimulation have been observed through classic extracellular recording (Fields et al., 1983). ON cell firing increases and OFF cell firing decreases, while Neutral cells exert no responses to nociceptive stimulus (M. M. Heinricher et al., 1987). Interestingly, other studies have suggested that Neutral cells could participate in the development of hyperalgesia by responding in an ON/OFF manner when applying noxious stimuli other than tail heat (Ellrich et al., 2000). Thus, ON and OFF cells are pivotal for RVM-related pain modulation.

Although studies have characterized the key role of the RVM in pain modulation, there are still several questions that remain unresolved in RVM-related pain modulation of neural networks, which impede the development of new analgesic approaches targeting the RVM and the corresponding pathways. These limitations include, but are not limited to: 1) neural circuits and neurotransmitters influencing the activities of RVM ON and OFF cells have not been precisely elucidated; 2) neurotransmitters participating in RVM ON and OFF cells driven spinal nociceptive modulation remain elusive.

In reality, the RVM is an integral relay point between high-level nerve systems and peripheral inputs in pain modulation. Nociceptive information is transmitted to the RVM through specific groups of spinal ascending neurons (Todd, 2010) and through connective neurons in supraspinal structures involved in pain modulation. The RVM receives nociceptive-related neural afferents, including the parabrachial complex (PB) (Roeder et al., 2016), locus coeruleus (LC) (Tanaka et al., 1996), midbrain (PAG) (Behbehani and Fields, 1979), hypothalamus (Gu and Wessendorf, 2007), and the amygdala (Mcgaraughty and Heinricher, 2002), and then projects to the trigeminal nucleus caudalis and the spinal dorsal horn via ventrolateral funiculus and spinal dorsolateral funiculus separately (Fields et al., 1995). Several key neurotransmitters have been demonstrated to be involved in RVM-related pain modulation, including opioids (Cheng et al., 1986; Lau et al., 2020), amino acids (Winkler et al., 2006), serotonin (Wei et al., 2010), and cannabinoids (Meng et al., 1998), which will also be discussed in this review. By providing an overview of the potential neural circuits and neurotransmitters corresponding to RVM ON and OFF cells, this review will deepen our current understanding of RVM-driven descending pain modulation.

## 2 Function and molecular characteristics of RVM ON and OFF cells

### 2.1 Functional definition of ON and OFF cells

According to cell discharge patterns and electroexcitation reactions in response to tail heat stimulation (Fields et al., 1983), Fields first confirmed that pronociceptive ON cells increased activity immediately before facilitation of the withdrawal response. Antinociceptive OFF cells decreased their relatively high spontaneous activity up to a pause, which triggered the nociceptive response. Neutral cells did not respond to painful stimuli. ON and OFF cells exert different activities and pain modulation effects under different circumstances. Under normal conditions, ON and OFF cells exhibit alternating periods of activity and silence (Barbaro et al., 1989). These synaptic interconnections within the RVM play an important role in modulating pain. Within the RVM, opioids directly inhibit the activities of ON cells, while indirectly increasing the activities of OFF cells, exerting analgesic effects (Cheng et al., 1986). Furthermore, when ON cells are inhibited and OFF cells are activated, this leads to responses of attentional analgesia (Oliva et al., 2021), placebo analgesia (Crawford et al., 2021), and stress-induced analgesia (Martenson et al., 2009). In the presence of nerve injury (Lopez-Canul et al., 2015) and in diabetic models (Silva et al., 2013; Palazzo et al., 2022) of hyperalgesia, increased ON cell activities and decreased OFF cell activities are observed, sustaining central sensitization and neuropathic pain responses. Therefore, any change in the balance of activity between ON and OFF cells produces bidirectional stimulatory or inhibitory modulatory effects on pain, regardless of normal circumstances or neuropathic and inflammatory situations.

### 2.2 Molecular expression characteristics of ON and OFF cells

Notwithstanding the electrophysiological properties of ON and OFF cells having been thoroughly investigated, the lack of specific cell markers encourages speculation into the neurotransmitters and receptors expressed by ON and OFF cells.

The RVM is considered the main site for opioid analgesia and opioid receptors are abundantly expressed in the RVM, including the μ-opioid receptor (MOR), κ-opioid receptor (KOR), δ-opioid receptor (DOR), and nociception/orphanin FQ receptors (NOPR), with especially high expression of MOR on ON cells. Previously, MOR has been considered a specific marker for ON cells (M. M. Heinricher et al., 1992). After injection of the micro-opioid MOR agonist DAMGO into the RVM *in vivo*, ON cell firing is directly inhibited through a postsynaptic mechanism (M. M. Heinricher et al., 1994). Indeed, MOR are positively expressed on part of OFF cells (Harasawa et al., 2016), and it has been assumed that the MOR agonists DAMGO activated OFF cells predominantly through two distinct mechanisms: one was direct activation via inducing glutamate release, while the other involved indirect disinhibition of RVM OFF cells by pre-synaptic inhibition of GABAergic terminals with the resulting release of endogenous opioids (M. M. Heinricher et al., 1992; M. M. Heinricher et al., 1994). KOR has limited expression on ON cells (approximately 13%) (Winkler et al., 2006). RVM microinjection of the KOR agonist U69593 did not affect tail flick latencies, although it did attenuate the ON cell burst (Meng et al., 2005), suggesting that OFF cells play a significant role in analgesia medicated by KOR. Approximately 86% of OFF cells are positive for KOR expression (Winkler et al., 2006), while functionally, administration of KOR agonists U69,593 into the RVM directly suppressed OFF cells activity through a postsynaptic mechanism and inhibited glutamatergic input to OFF cells presynaptically, and thus could elicit mechanical hypersensitivity under normal conditions or following injuries (Winkler et al., 2006; Lau et al., 2020). Furthermore, KOR was found to be necessary and sufficient to mediate stress induced analgesia (SIA) since activation of KOR exerted similar effects as SIA, which were not enhanced by stress (Bagley and Ingram, 2020). Moreover, Nguyen et al. (2022) demonstrated that chemogenetic activation of KOR-positive neurons in the RVM increased thresholds of heat and mechanical pain and reversed hypersensitivity in acute and chronic pain states, which was consistent with presynaptic inhibition of excitatory inputs to ON cells and direct facilitation of OFF cells activity. Taken together, these results strongly indicate that KOR is expressed mainly on OFF cells but not on ON cells. After microinfusion of the DOR agonist DELT, ON cells activities decrease, while OFF cells activity is increased and behavioral antinociception is observed (Harasawa et al., 2000). Similar to MOR, the activation of DOR inhibits ON cells directly, disinhibits OFF cells, and induces analgesia. NOPR is co-expressed with MOR on ON cells, and both agonists exhibit the same suppression effects on ON cells, although the effects are not a crucial step in opioid-mediated analgesia (M. M. Heinricher et al., 1997). Previous studies have shown that under the circumstances of naloxone-precipitated withdrawal from opioids, infusing NOPR inhibited ON cell activity, removed the pronociceptive influence and counteracted the opioid withdrawal-induced hyperalgesia (Kaplan and Fields, 1991; Pan et al., 2000). Interestingly, NOPR activity induces pain-increasing effects via reversing the disinhibitory effect of facilitating OFF cells in the presence of stress stimulation, in which KOR participated (Pan et al., 2000). Thus, endogenous opioids interact with both ON and OFF cells, which fail to be identified as specific markers of any cell groups.

The effects of GABA and glutamate on RVM ON and OFF cells cannot be overlooked, as their dysfunction may represent a specific marker of ON or OFF cells. Approximately two-thirds of RVM ON cells contain express γ-aminobutyric acid (GABA) (Winkler et al., 2006). However, after administration of the GABA_A_ receptor antagonist bicuculline methiodide, analgesic effects were observed, although ON cells did not show a consistent change in activity (M. M. Heinricher and Tortorici, 1994). Further research is still needed to determine whether other GABA receptors mediate ON cell modulation. Interestingly, RVM GABA transporter positive (VGAT^+^) GABAergic neurons express immune-responsive MOR (MOR-ir), and approximately 67% of MOR-ir RVM neurons are vesicular VGAT^+^ (Francois et al., 2017), indicating co-expression of MOR and GABA in ON cells. Activation of MOR or DOR produces a concentration-dependent decrease of GABA overflow in the RVM, reduces inhibitory GABAergic activity, and directly hyperpolarizes ON cells (Schepers et al., 2008; C. Zhang et al., 2020). Meanwhile, recent research by Jiao et al. (2022) confirmed that the G protein-coupled estrogen receptor (GPER) was expressed specifically in GABAergic ON cells, as activation of GPER caused depolarization of ON cells through modulation of MOR and potentiated pain. These findings may explain the major role of GABA and MOR in ON cell modulation, although GPER are found to be specific only in GABAergic groups. Furthermore, GAD67 (GABA markers) were identified in approximately 93% of OFF cells by immunostaining, indicating that OFF cells are also GABAergic neurons (Winkler et al., 2006). Since only 22% of GABA_B_-expressing neurons in the RVM were retrogradely labeled, activating GABA_B_ receptors at low doses facilitates OFF cells, while these were inhibited at high doses (Pinto et al., 2008). Following administration of the GABA_A_ receptor antagonist bicuculline, OFF cells enter a prolonged period of continuous firing and exert behaviorally measurable antinociception (M. M. Heinricher and Tortorici, 1994), reiterating the importance of the OFF cell disinhibition on GABA_A_ receptor-induced antinociception.

Regarding glutamate, repeated intramuscular injections increase the response of ON cells to glutamate by altering the activity of the N-methyl-D-aspartate receptor (NMDA), a glutamate receptor (Da Silva et al., 2010). By microinjecting the NMDA receptor antagonist AP5 into the RVM, Xu et al. (2007) found that only ON cell activities were inhibited, while activated OFF cells and Neutral cells were not affected, exerting a block of hyperalgesia. By applying the NMDA receptor antagonist ketamine to the RVM, ON cells were inhibited, which caused a reversal of chronic morphine-induced heat hypersensitivity (Viisanen et al., 2020). Moreover, the injection of low-dose glutamate into the RVM facilitated nociception in models of neuropathic and inflammatory pain, where the expression of cannabinoid receptor 1 (CB1R) and NMDA/AMPA receptors (glutamate receptors) on RVM ON cells was increased (Radhakrishnan and Sluka, 2009; Boadas-Vaello et al., 2016; Li et al., 2017). In general, we believe that NMDA receptors and glutamate in RVM could enhance ON cell activities and produce hyperalgesia. Conversely, OFF cells exert stronger activity and produce antinociceptive effects following application of glutamate or the specific activation of metabotropic receptors 8 (mGluR8) (Hosseini et al., 2021). Consequently, infusing the D-2-amino-5-phosophonopentanoic acid (NMDA) receptor antagonists into the RVM could attenuate or block the activation of OFF cells, which congruently confirmed the expression of glutamate receptors on OFF cells (M. M. Heinricher et al., 2001).

The transient receptor potential vanilloid-1 (TRPV1) is also expressed by both ON and OFF cells. Through immunoreactivity studies, Starowicz et al. (2007) found that in naïve rats, TRPV1/MOR was colocalized in 84% ± 5% of RVM neurons, while 21% ± 3% of TRPV1-labeled cells were surrounded by positive nerve terminals for VGAT, and of these, 80% ± 7% also expressed vesicular glutamate transporter 1 (VGLUT1). As MORs are mainly expressed in ON cells, we prefer to consider the existence of TRPV1 in ON cells under normal conditions. Interestingly, in the streptozotocin (STZ)-induced diabetic rat model, administering the capsaicin agonist TRPV1 increased TRPV1 expression and decreased hyperalgesia in the formalin test, with no effect in control animals (Silva et al., 2016). It is reasonable to suggest that during diabetic neuropathy, rats tend to be more sensitive to modulation of TRPV1 activity and changes in TRPV1 expression may be involved in glutamatergic circuits, namely OFF cells (Maione et al., 2009).

Microinjection of cholecystokinin (CCK) at low doses (10 ng/200 nL) attenuated opioid activation of OFF cells and blocked opioid-mediated analgesia; however, it exerted no direct effects on ON or OFF cells (Heinricher, 2001). Only administration of CCK at higher doses (30 ng/200 nL) selectively activated ON cells and produced behavioral hyperalgesia (M. M. Heinricher and Neubert, 2004). Therefore, the anti-opioid and pronociceptive effects induced by CCK within the RVM are mediated by OFF and ON cells separately. Furthermore, Zhang et al. (2009) confirmed that 80% of RVM neurons co-expressed MOR and the CCK type 2 receptor (CCK2), and that MOR was specifically expressed in ON cells. After microinjecting L365,260 (a selective antagonist for CCK2) rather than a CCK1 antagonist into the RVM, the thermal hypersensitivity of rats with spinal injury was reversed (Kovelowski et al., 2000). Meanwhile, in female rats after plantar incision, injecting the CCK2 receptor agonist CCK-8 into the RVM produced hyperalgesia, while the CCK2 receptor antagonist YM022 decreased sensitivity to pain (Jiang et al., 2019). These studies provide evidence that CCK acts through CCK2 on ON cells and contributes to hyperalgesia under normal and pathological conditions. However, it remains unclear whether CCK2 can be regarded a specific marker of ON cells, as there has been no study investigating whether all ON cells express CCK2 and the direct effects on ON or OFF cells when facilitating or inhibiting CCK2.

Immunohistochemical studies have shown that the neurokinin-1 receptor (NK-1R) of substance P (SP) is co-expressed with NMDA receptors in RVM ON cells (Budai et al., 2007). Activation of NK-1R through microinjection of the NK-1R agonist SP or capsaicin actually improved ON cell responses evoked by NMDA receptors but not those of OFF cells and promoted hyperalgesia; these changes were attenuated by iontophoretic application of the NK-1R antagonist L-733,060 (Budai et al., 2007). However, NK-1R cannot be considered a specific marker of ON cells, although SP and NK-1R have been confirmed to promote descending facilitation and contribute to central sensitization and hyperalgesia. First, NK-1 positive neurons were a relatively small proportion of all neurons in the RVM, therefore, it is not clear whether all ON cells express NK-1R. Moreover, in the absence of inflammation, injecting L-733,060 or SP fails to change mechanical antinociception but alters thermal antinociception (Hamity et al., 2010). Meanwhile, pretreatment with L-733,060 in the RVM decreases nociception sensitivity only after injecting capsaicin, rather than complete Freud’s adjuvant (CFA) (Hamity et al., 2010; Brink et al., 2012), indicating its limit in the processing of different pain stimuli (Khasabov et al., 2012).

**FIGURE 1:**
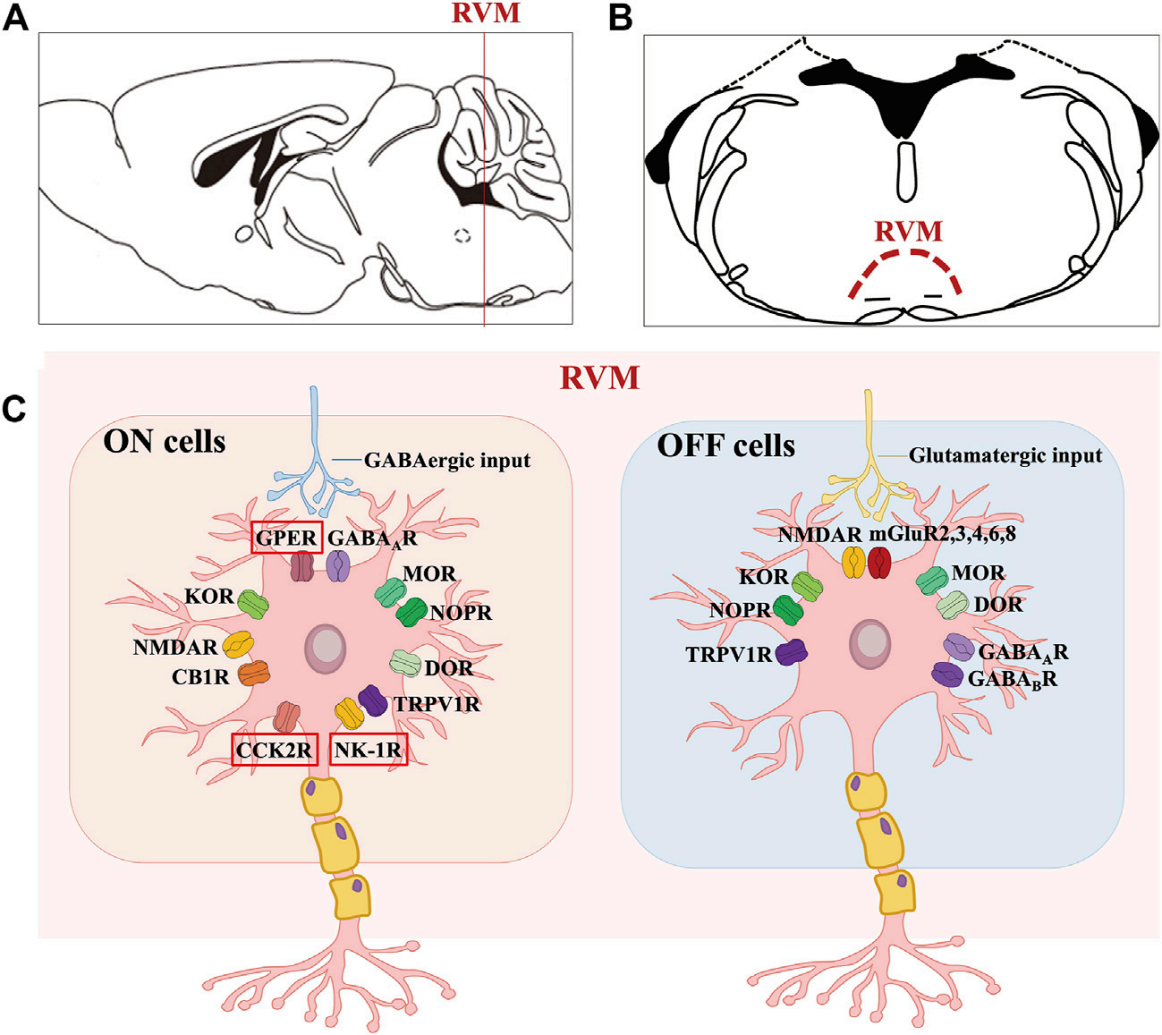
Molecular characteristics of the ON and OFF cells in RVM. **(A, B)** shows the anatomical location of the RVM nucleus in the mouse brain. **(C)** shows that GPER along with opioid, cannabinoid, GABA, TRPV1, glutamate, NK-1 and CCK2 receptors are expressed on ON cells, where GPER, CCK2R and NK-1R may be specific. Likewise, OFF cells mainly express opioid, cannabinoid, GABA, TRPV1 and glutamate receptors.

Neurotensin (NT) is mainly expressed in the RVM and exerts nociceptive or antinociceptive effects in different states of pain (Feng et al., 2015). The low dose of NT in the RVM selectively activates the CCK2R in ON cells, increases the release of CCK, and produces thermal hyperalgesia, while high doses of NT recruit OFF cells and activate NT receptors, resulting in antinociception (Neubert et al., 2004; Li et al., 2021). Both the high-affinity NT receptor (NTR1) and the low-affinity NT receptor (NTR2) are expressed in RVM, of which NTR1 is predominantly co-expressed with 5-HT, whereas NTR2 is rarely expressed in 5-HTergic neurons. Antinociception produced by PD149163 (NTR1 agonists) is blocked by methysergide (non-selective serotonergic receptor antagonists) and is partially blocked by intrathecal yohimbine (NA receptor antagonists), although β-LT (NTR2 agonists) induced antinociception is only inhibited by yohimbine (Buhler et al., 2005; Buhler et al., 2008; Li et al., 2021). Taken together, it is plausible that NTR1-mediated antinociception is mediated by noradrenaline (NA) and 5-HT release, while NTR2-mediated antinociception involves spinal release of NE alone. For the aim of pain relief, we conclude that through activating MOR, DOR, GPER and GABA, or inhibiting KOR, NMDA, TRPV1, CCK2R, CB1, NK-1R and NTR1, ON cells firing can be inhibited and OFF cells firing can be facilitated, leading to analgesia (Figure 1).

Although the role of neutral cells in pain modulation remains unknown, studies have suggested that they could develop ON or OFF cell-like properties under pathological conditions (Ellrich et al., 2000; Miki et al., 2002). Approximately 80% of neutral cells express GAD67, with a similar proportion expressing KOR (Winkler et al., 2006). In the RVM, approximately 23% of all neurons were found to be serotoninergic (5-HTergic), among which the vast majority were Neutral cells, while slightly fewer were OFF cells and the least were ON cells (Gu and Wessendorf, 2007; Lima et al., 2017). Serotonin (5-HT) has also been found to be expressed exclusively in a subset of neutral cells (36%), while none of ON or OFF cells exhibit 5-HT immunoreactivity (Winkler et al., 2006; Wang et al., 2017). It is worth paying closer attention to the exact function of 5-HTergic neutral cells in chronic pain modulation.

## 3 Neural circuits and neurotransmitters modulating RVM ON and OFF cells

### 3.1 Periaqueductal gray

#### 3.1.1 Neural circuits involved in PAG-driven descending modulation of RVM

As the first discovered nuclei modulating descending pain in the brain, the periaqueductal gray (PAG) plays a key role in modulating descending pain, with direct projection to the RVM, and this projection terminates in superficial laminae (laminae I and II) of the spinal dorsal horn (Fields and Basbaum, 1978; Lau et al., 2020). Projections from PAG to RVM ON and OFF cells react to noxious stimuli and generated protective or defensive effects on pain sensation as defined using animal studies (M. M. Heinricher et al., 1987), which were also clearly confirmed by human brain imaging studies (Oliva et al., 2021). In-depth observations further provided stronger evidence indicating that opioid-triggered analgesia was mediated by ventrolateral projections of the PAG (vlPAG)-RVM, while non-opioid-triggered analgesia could be aroused by projections of the lateral PAG and the dorsolateral PAG (lPAG/dlPAG)-RVM (Crawford et al., 2021). As there have been no subsequent investigations, the delicate circuit constructions from the PAG to RVM for pain modulation are still far from being clarified.

#### 3.1.2 Neurotransmitters mediating PAG-driven descending modulation of RVM

As the descending pain modulation circuit of the brain, the transmitters in the PAG-RVM projections have been comprehensively studied and include mainly GABA, glutamate, opioid, cannabinoid, and dopamine (DA) neurotransmitters. The vlPAG has direct GABAergic projections to RVM ON and OFF cells. Through immunochemistry studies, approximately 71% of the synapses in the PAG appeared to be GABAergic (containing GAD67 immunoreactivity), and directly projected to the RVM ON, OFF, and Neutral cells (Morgan et al., 2008). Microinjecting the GABA_A_ receptor antagonist bicuculline into vlPAG, the spontaneous activity of OFF cells increased while that of ON cells decreased, resulting in pain inhibition (Moreau and Fields, 1986). When exposed to repeated restraint stress, GABA release into the RVM was increased, facilitating ON cells activity and inhibiting OFF cells activity, hence leading to mechanical hypersensitivity (Kohn et al., 2020). Several studies have suggested that GABA plays a major role in opioid and cannabinoid induced analgesia through the PAG-RVM pathway and is discussed below.

Glutamate also plays an important role in pain modulation via the PAG-RVM pathway with a pattern similar to that of GABA. Ho et al. (2013) found that when exposed to nerve injury, the α-amino-3-hydroxy-5-methyl-4-isoxazolepropionic acid (AMPA, another ionotropic transmembrane receptor for glutamate) receptor response was decreased, while the response of the NMDA receptor increased in vlPAG, decreasing the activities of OFF cells, thus initiating and facilitating hyperalgesia. Furthermore, the researchers identified eight subtypes of glutamate metabotropic receptors (mGluR1-8) in PAG exert different effects on nociception modulation, of which hyperalgesia was elicited by activating mGluR1 and mGluR5 (Group I), while activation of mGluR2, mGluR3 (Group II), and mGluR4, 6, 7, 8 (Group III) led to analgesia. Studies conducted by Hosseini et al. (2020) showed that after administration of mGluR8 agonists (S)-3,4-dicarboxyphenylglycine (DCPG) in PAG, glutamate transmission increased, then RVM ON cell firings were reduced and OFF cell activities were enhanced, and predominantly generated antinociceptive effects. However, Marabese et al. (2007) reported a disturbing finding in which intra-PAG microinjection of AMN082, a selective mGluR7 agonist, increased RVM ON cell activity while abrogating OFF cell activity and then induced hyperalgesia. It is plausible that hyperalgesia was attributed to the activation of mGluR7 in the PAG before that of mGluR4/8, which overlaps the antinociceptive effects triggered by mGluR4/8.

Opioid mediated analgesia mainly through PAG-RVM circuit. Via the presynaptic inhibition of PAG GABAergic interneurons and glutamatergic transmission, the microinjection of morphine into the PAG inhibited GABAergic projections to the RVM (Beitz, 1990; Park et al., 2010; Lau et al., 2020), indirectly activated (disinhibited) RVM OFF cells and directly inhibited ON cell activity, thereby leading to analgesia (Basbaum and Fields, 1984; M. M. Heinricher et al., 1987; Fang et al., 1989; M. M. Heinricher et al., 1994). Although opioids released from PAG to RVM play a pivotal role in endogenous analgesia, more in-depth studies are needed to clarify the expression of different opioid receptors in this system. Similar to the observations in the RVM, MOR, DOR, KOR, and NOPR were also prevalent in the PAG region (Gutstein et al., 1998). MOR expression in the PAG is located primarily in GABAergic projection neurons to the RVM (Commons et al., 2000). Vaughan et al. (1997) found that MOR coupled to a voltage-dependent K+ conductance in the GABAergic terminals via the PLA2/arachidonic acid/12-lipoxygenase cascade system, inhibits GABAergic interneurons in the PAG. G protein-coupled inwardly rectifying potassium channels (GIRKs) were also found to be activated by MOR, leading to hyperpolarization of PAG-RVM projections (Morgan et al., 2020). These findings enhanced the reliability of the disinhibition theory. Regarding DOR, the mechanisms of pain modulation and the effects of DOR in the PAG-RVM circuit were similar to MOR, except for the indirect inhibition of GABAergic projections (Kalyuzhny and Wessendorf, 1998). Accumulating evidence suggests that DOR is located mainly in terminals containing enkephalins of the PAG, and when released, glutamatergic inputs to GABAergic projections in the vlPAG would be inhibited, including the metabolic pathways involving CB1R and phospholipase A (Commons et al., 2001; Z. Zhang and Pan, 2012; Bushlin et al., 2012). NOPR can presynaptically inhibit GABAergic and glutamatergic neurons in the vlPAG and postsynaptically inhibit PAG-RVM projections, leading to hyperalgesia and reverse opioid-induced analgesia (Morgan et al., 1997; Vaughan et al., 1997; Lu et al., 2010).

Interestingly, the PAG-RVM opioidergic projections are closely related to the sexual dimorphism of pain modulation. Studies have shown that antinociceptive effects of morphine were reduced in intact gonadal females compared to males, while MOR and KOR agonists were more potent or effective in one sex or the other in more than 60% of cases (Craft, 2003). Additionally, Tonsfeldt et al. (2016) observed that antagonists or negative allosteric modulators of the GABA_A_ receptor appeared to be more efficient in morphine-induced analgesia of female rats compared to that of male rats during continuous inflammation. Therefore, it is reasonable to suspect that sexually dependent neurotransmitters may modulate opioid-mediated analgesia. Craft et al. (2004) suggested that compared to males, morphine-mediated antinociception could be attributed at least in part to higher levels of estradiol in females, since in female rats the baseline ON cell burst and OFF cell pause were depressed. Meanwhile, researchers found that estrogen receptor alpha was required for estrogen-induced internalization of MOR (Micevych et al., 2003). Loyd et al. (2007) further demonstrated that the PAG-RVM projections contained less MOR immunoreactivity in vlPAG in females compared to males, resulting in the attenuation of opioid-mediated analgesia. Furthermore, reproductive hormone levels have been found to influence the distribution of KOR in RVM, which, in turn, modulates opioid-mediated analgesia (Drake et al., 2007). All of these results have confirmed the crucial role of the opioid system in PAG-RVM projections to mediate pain-related sexual dimorphism.

Endocannabinoid also participates in analgesia through PAG-RVM pathway. The endocannabinoid system consists of the endogenous synthesis enzyme lipid signals 2-arachidonoylglycerol and the degraded enzyme anandamide, and also the cannabinoid receptors. Except in some cortex regions, PAG is a key nucleus that synthesizes endocannabinoids, which are in turn released to the RVM. The projections of the PAG-RVM mediate cannabinoid-induced analgesia and contribute to SIA (Ossipov et al., 2010). Microinjection of cannabinoid receptor agonists WIN55,212-2 into the RVM decreased the firing of ON cells while increased ongoing OFF cells activities, thus increasing the rat tail-flick latency (Rea et al., 2007; Ossipov et al., 2010). Therefore, the endogenous cannabinoids can modulate the tonic increase in OFF cells activity and diminish ON cells firing, modulating baseline nociceptive thresholds and exerting antinociceptive effects. Cannabinoid receptors consist of cannabinoid receptor 1 and 2 (CB1R, CB2R), among which CB1R is expressed in approximately one-third of PAG neurons and is co-expressed with MOR. Activation of PAG CB1R decreases GABA release and activates mGlu5R, leading to the inhibition of ON cells and disinhibition of OFF cells, ultimately resulting in SIA, as well as analgesia in both normal and neuropathic pain situations (Novellis et al., 2005; Rea et al., 2007; Ossipov et al., 2010). However, CB2R expression appears to be highly dynamic and depends on the microenvironment, since CB2R expression increases in inflammation and neuropathic pain (Bouchet and Ingram, 2020). Through *in vivo* recording assays, the CB1R agonists SR141716 activated OFF cells, which was consistent with their analgesic effects on pain behavior, while through *ex vivo* slice recording, SR141716 significantly decreased the frequency of miniature inhibitory postsynaptic potential (mIPSC, related to GABA release) frequency and inhibited GABA release to ON cells (Li et al., 2015; Bouchet and Ingram, 2020; Milligan et al., 2020). Of note, CB2R agonists AM1241 and GW405833 inhibited presynaptic GABA release and ON cell activities in the RVM in CFA-treated but not in naïve rats (Bouchet and Ingram, 2020). Alternatively, it has been fully demonstrated that endogenous cannabinoids activate opioid-insensitive SIA predominantly through the CB1R rather than the CB2R, and inhibiting endogenous cannabinoids hydrolysis in the RVM can enhance SIA (Ossipov et al., 2010).

Compared to opioid-related analgesia, the contribution of PAG dopaminergic neurons (DAergic) to pain modulation via the vlPAG-RVM pathway is obscure. To our knowledge, there are indeed DAergic neurons in vlPAG, but no direct DAergic projections from the PAG to RVM (Li et al., 2016). However, DA may activate the dopamine receptor 2 (D2R), which was expressed in PAG GABAergic neurons (Li et al., 2016; Ferrari et al., 2021). Meyer et al. (2009) determined that injection of the D2R agonist quinpirole but not dopamine receptor 1 (D1R) agonists chloro-APB into the vlPAG modulated PAG-RVM projections, and increased the threshold for the paw-withdrawal response and generating protective reactions to pain, while suppressing DAergic neurons reduced opioid-mediated analgesia (Ferrari et al., 2021). Li et al. (2016) determined D2R activation blocked MOR-induced inhibition of GABAergic neurons and reduced presynaptic GABAergic neurotransmission, leading to a decrease in inhibitory input to vlPAG DA neurons and antinociception. In contrast, the administration of D-amphetamine to vlPAG led to an inhibition of RVM ON cells by PAG GABAergic neurons, although increased PAG glutamatergic projections to RVM OFF cells were not involved in antinociception effects (Ferrari et al., 2021). Collectively, it has been speculated that the analgesic effects of PAG DA neurons are mediated by indirect modulation of the RVM, mainly by interfering with the opioid- and GABA-mediated descending pathway of PAG-RVM.

**FIGURE 2:**
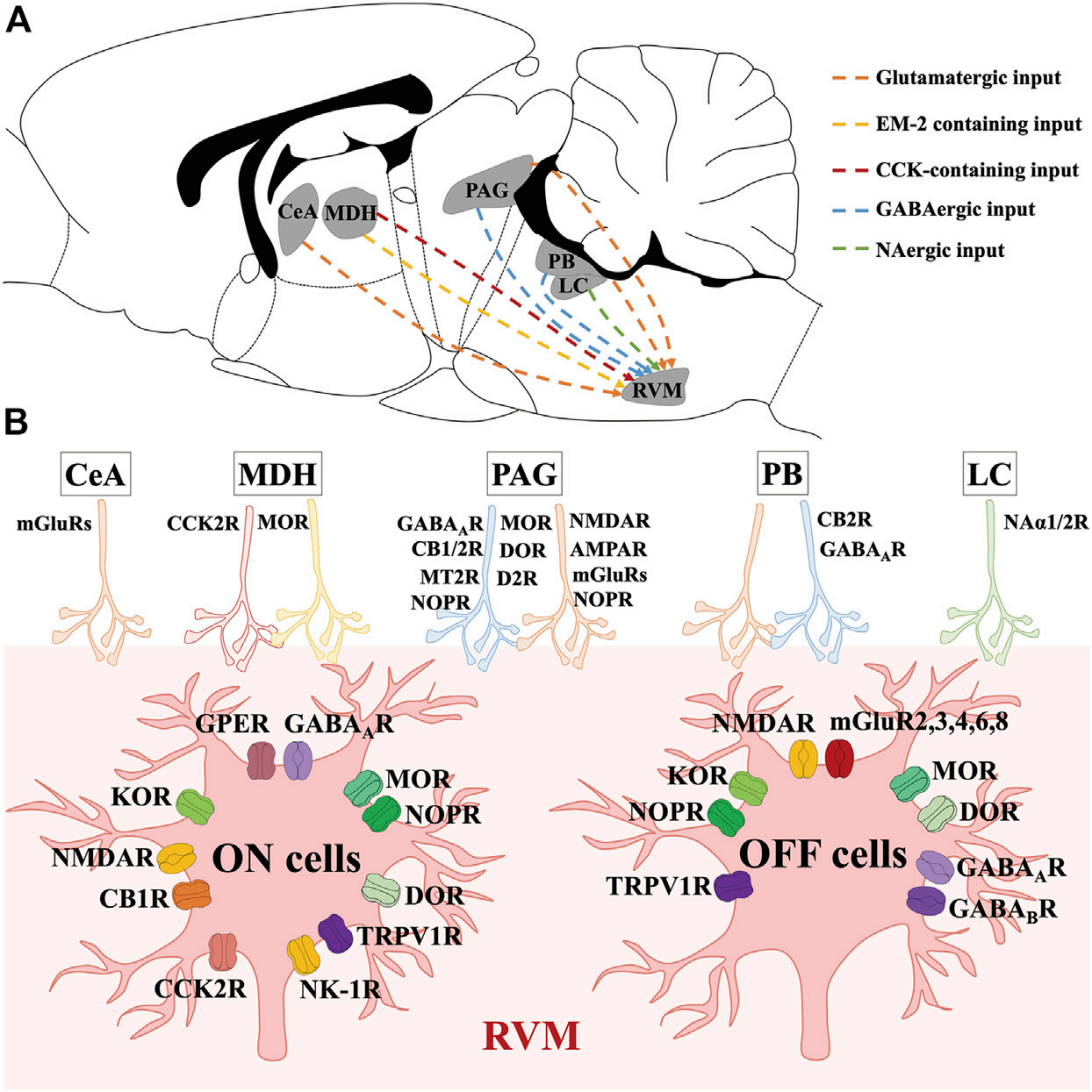
Molecular characteristics of neural circuits and transmitters modulating RVM ON and OFF cells. **(A)** shows the anatomical location of nucleus and neural circuits projecting to the RVM ON and OFF cells in the mouse brain. **(B)** shows the transmitters and receptors which participate in modulating RVM ON and OFF cells. CeA mainly act on glutamate receptors to exert modulations on RVM ON and OFF cells. By binding to MOR and CCK2R, DMH EM-2 containing inputs and CCK-containing inputs modulate RVM ON and OFF cells. Via acting on opioid, cannabinoid, GABA, glutamate, dopamine, TRPV1, and melatonin receptors, PAG GABAergic and glutamatergic inputs modulate RVM ON and OFF cells. PB GABAergic and glutamatergic neurons modulate RVM ON and OFF cells by binding to cannabinoid, and GABA receptors. LC NAergic neurons act on Naα receptors to modulate RVM ON and OFF cells.

Melatonin (MLT) participates in analgesia across different states of abnormal pain. Specifically, MLT acts primarily through melatonin 2 (MT2) receptors in vlPAG, inhibiting ON cell activities and facilitating OFF cells, leading to analgesia (Lopez-Canul et al., 2015). Interestingly, researchers found that only 0.2% of MOR was co-expressed with melatonin 2 (MT2) receptors in vlPAG, but all MT2 receptors, which did not show expression in RVM, required MOR to exert antinociceptive effects (Posa et al., 2022) (Figure 2).

### 3.2 Parabrachial complex

#### 3.2.1 Neural circuits involved in PB-driven descending modulation of RVM

The parabrachial complex (PB) functions as a significant supraspinal relay region in the midbrain for nociceptive transmission, not only under normal situations but also under pathological pain conditions. PB was shown to project directly to the RVM using bulk tracer methods (Verner et al., 2008). Interestingly, electrophysiological investigations determined that during the PB block, the extent of OFF cell pauses and ON cell bursts was attenuated, but not eliminated, suggesting a modest rather than robust decisive contribution to modulation to RVM ON and OFF cell activities in the pain process. Additionally, optogenetic inhibition of archaerhodopsin (ArchT) expressing PB terminals in the RVM can substantially attenuate ON cell (62%) and OFF cell (71%) activity during *in vivo* recording, leading to antinociception (Chen et al., 2017). Furthermore, blocking contralateral PB, but not ipsilateral PB, significantly attenuated the burst of ON cells and the pause of OFF cells in the first hour after CFA injection and substantially reversed mechanical hypersensitivity, while during the ∼1–6 days after CFA injection, blocking the contralateral PB did not alter the firing of ON or OFF cells and did not exert any effects on hyperalgesia. In contrast, during the ∼5–6 days after CFA administration, only activating the ipsilateral PB interfered with the evoked responses in the RVM, which resulted in an increase in overall ON cell firing and a reduction in OFF cell activities, exacerbating the inflamed hind paw, and ultimately improving and maintaining pain hypersensitivity (Chen and Heinricher, 2019b). Altogether, the general consensus indicates that PB receives substantial projections from nociceptive neurons in the contralateral superficial dorsal horn, and less dense inputs from the ipsilateral superficial dorsal horn and deeper lamina, more specifically, the PB projects directly to RVM ON and OFF cells. Under physiological conditions and when exposed to acute pain stimuli, it is the contralateral PB that transmits projections to the RVM ON and OFF cells and then triggers acute hyperalgesia, while the ipsilateral PB is involved in the RVM ON and OFF cells-induced modulation of persistent inflammation and chronic pain.

#### 3.2.2 Neurotransmitters mediating PB-driven descending modulation of RVM

PB-RVM circuits exert hyperalgesia mainly through GABAergic and glutamatergic projections. Most PB neurons were glutamatergic (72%) or GABAergic (28%) (Geerling et al., 2017; Chiang et al., 2019). In particular, by whole cell patch clamp recordings, Chen et al. found that light stimulation of PB neuron terminals that expressed ChR2 in RVM slices elicited synaptic currents in 29 neurons, of which 21 neurons were blocked by glutamate antagonists KA or NBQX, while the remaining 8 neurons were inhibited by bicuculine, a GABA_A_R antagonist. Furthermore, the GABAergic inputs observed in slice experiments formed inhibitory nociceptive inputs to OFF cells, while the glutamatergic inputs corresponded to excitatory projections to ON cells and some were concomitant with Neutral cells (Chen et al., 2017). Importantly, under stress, lPB neurons, specifically those projecting onto RVM GABAergic neurons (that is, ON cells), cooperate with cannabinoids and CB2R and participated in hyperalgesia (Francois et al., 2017) (Figure 2).

### 3.3 Locus coeruleus

As an important mental stress-responsive nucleus, the locus coeruleus (LC) is also a pivotal region involved in the modulation of descending pain, mainly through direct spinal cord projections and effects on RVM activity via NAergic projections. Through a retrograde tracing study, Braz et al. (2009) found that ventrally located LC neurons were labeled after injecting pseudorabies virus (PRV) into the RVM, which is consistent with the results that in attentional analgesia, an fMRI parameter of LC-RVM connections was improved (Tanaka et al., 1996; Hickey et al., 2014; Oliva et al., 2022). Moreover, studies by Dehkordi. et al., 2019 demonstrated that stimulation of LC NAergic neurons increased the release of NA and increased 1-adrenoceptor concentrations of α1-adrenoceptors (NAα1R) in NRM, leading to analgesia (Dehkordi. et al., 2019). In contrast, it was also possible that LC-NRM projecting NAergic neurons induced hyperalgesia by activating NRM NAα1R. As Bie et al. (2003) showed, during opioid withdrawal, administration of the NAα1R antagonist prazosin in NRM significantly decreased the activities of NAergic projections of the LC and suppressed hyperalgesia. RVM ON cells received NAergic inputs from LC and contained NAα1R, which contributed to hyperalgesia during opioid withdrawal, while inhibition of NAα2R-expressed ON cells by clonidine suppressed DAMGO-mediated analgesia instead of modulating hyperalgesia during opioid withdrawal. OFF cells also received dense NAergic input from the LC, and mainly expressed NAα1R and some co-expressed NAα2R (Bie et al., 2003). Overall, these results suggest that LC-RVM projections on NAergic neurons trigger not only antinociceptive but also pronociceptive effects by modulating RVM ON and OFF cells, although the precise role of LC-RVM projections in different pain conditions requires further investigation (Figure 2).

### 3.4 Hypothalamus

Hypothalamus-RVM circuits participate in pain modulation mainly in dorsal medial hypothalamus (DMH) via endomorphin-2 and CCK containing projections. After microinjection of Fluoro-Gold (FG) into the RVM, retrogradely labeled neurons were detected in the hypothalamus, the majority of which were present in the lateral hypothalamus (LH) and DMH (Gu and Wessendorf, 2007). DMH stimulation directly induced robust activation of ON cells along with suppression of OFF cell firing, leading to behavioral hyperalgesia, in contrast to other research showing that stimulation of the hypothalamus produced analgesia that could be inhibited by systemic naloxone (Adams and Hosobuchi, 1977; Martenson et al., 2009). Hyperalgesia induced by DMH stimulation recruits ON cells under mild and persistent stress, a response known as stress-induced hyperalgesia (SIH). However, microinjection of lidocaine into the RVM potentiates hypothalamic-mediated analgesia, as observed by the increase in the pain threshold and inhibition of the tail flick reflex (Gu and Wessendorf, 2007). Collectively, these findings confirm that the hypothalamus projects directly to the RVM and influences the activities of ON and OFF cells; thus, exerting bidirectional pain modulation. In terms of neurotransmitters and receptors, endomorphin-2 (EM-2, an endogenous ligand of MOR) containing neurons exist primarily in the DMH and project to the RVM, and EM-2 has been found to participate in hypothalamus stimulation-induced analgesia, replicating its antinociceptive effects through MOR and endogenous opioids (Gu and Wessendorf, 2007; Bagley and Ingram, 2020). Furthermore, in stress states, DMH has been identified as the only supraspinal source of CCK input to the RVM, acting with CCK2R in ON cells and can elicit hyperalgesia as observed experimentally under abundant abnormal pain states via retrograde tract tracing combined with electrophysiological and immunohistochemistry (Wagner et al., 2013). Moreover, infusion of 1 nmol excitatory amino acid receptor cytonurenate into the RVM blocked the DMH-induced ON cell activation and suppression of OFF cells, alleviating hyperalgesia. Similar results can also be observed after infusion of the GABA_A_ receptor agonist muscimol (Martenson et al., 2009). In conclusion, the hypothalamus-RVM projection modulates pain sensitivity primarily through endogenous opioid synaptic transmission and communicates with CCK2R and MOR (Figure 2).

### 3.5 Amygdala

The amygdala is characterized by direct and indirect projections to the RVM, and participates in pain modulation. After microinjection of morphine into different sites of the amygdala, several significant findings suggest that its analgesic effects were mainly attributed to the direct projections from the amygdala to the RVM, for instance: 1) infusing morphine into the basolateral nuclei increased OFF cell activity, modestly decreased ON cell activity, remarkably attenuated the OFF cell pause, and increased tail flick latency; 2) administering morphine into the cortical and medial nuclei exerted smaller effects on ON and OFF cells than infusion directly into basolateral nuclei, but did not significantly eliminate the OFF cell pause and only the increased tail flick latency to a small degree; 3) introducing morphine within the central, medial, and dorsal lateral nuclei failed to modulate activities of the RVM ON and OFF cells and the tail flick latency (Mcgaraughty and Heinricher, 2002). In fact, the basolateral nucleus of the amygdala is relayed through the central nucleus of the amygdala (CeA), and then recruited RVM OFF cells, and conducts opioid-induced analgesia under states of SIA to replenish DMH-induced SIA (Martenson et al., 2009). Despite the ineffective function of CeA under normal conditions, it is currently agreed that amygdala-mediated hyperalgesia in pain-related disorders occurs in CeA through the interactions with mGluR1/5, since there are large amounts of nociceptive neurons at this pivotal site and an increase in excitability of CeA is detected even under conditions of chronic pain (Palazzo et al., 2011). Meanwhile, CeA exerts antinociceptive effects by acting on the mGluR8. Under carrageenan-triggered inflammatory pain conditions, intra-CeA microinjections of mGluR8 agonists (S)-3,4-DCPG increase OFF cell activities while decrease ON cell activities, thus creating antinociceptive effects. (Palazzo et al., 2011). Hence, amygdala-RVM pathway, particularly CeA-RVM projections, modulate pain through acting at mGluRs (Figure 2).

## 4 Neural circuits and neurotransmitters modulating projections from RVM ON and OFF cells to spinal cord

### 4.1 Neural circuits involved in RVM-driven descending modulation

There exist RVM-spinal cord circuits modulating pain through 5-HTergic and GABAergic projections. It is generally accepted that descending projections from the RVM to the spinal cord predominantly target the laminae V dorsal horn (Martins and Tavares, 2017), although the axons of most spinal ascending projection neurons terminate in many areas of the brain, including the forebrain, pons, and midbrain, rather than in the RVM, suggesting that RVM does not receive afferents from the spinal cord (Wang et al., 2022). By injecting rabies virus into the spinal cord, Francois et al. (2017) used retrograded transsynaptic tracing to identify regions corresponding to descending pain control and found that the virus was strongly expressed in RVM neurons; thus, indicating that RVM projects to the spinal cord. Furthermore, more than 90% of the RVM spinal cord projection neurons responded to the opioid agonists DAMGO, of which a large proportion of the neurons responded only to MOR agonists (64%), while a smaller proportion responded only to KOR agonists (9%), and approximately 18% responded to both MOR and KOR agonists (Marinelli et al., 2002). In fact, the RVM descending projection neurons that participate in pain modulation mainly consist of GABAergic and 5-HTergic neurons, so how these inhibitory GABAergic neurons facilitate spinal pain transmission was previously a mystery. Francois et al. demonstrated that downstream of RVM GABAergic neurons were spinal GABAergic/enkephalinergic interneurons, and activation of the RVM GABAergic neurons would inhibit spinal inhibitory interneurons, thus inducing disinhibition of spinal pain transmission (Francois et al., 2017). The study revealed a potential circuit mechanism for ON cells-induced pain facilitation, although it is still urgent to understand how OFF cells and 5-HTergic neurons modulate spinal pain transmission and how these RVM neurons cooperate in different pain states.

### 4.2 Neurotransmitters mediating RVM-driven descending modulation

GABAergic projections from the RVM to the spinal cord take part in pain modulation through altering the activities of RVM ON and OFF cells. Most RVM neurons that project to the spinal cord are not 5-HTergic (60%, i.e., GABAergic), of these, 40% also express enkephalin (PENK), while the remaining 40% are 5-HTergic (Hossaini et al., 2012; Kohn et al., 2020; Talluri et al., 2022). The RVM GABAergic neurons express GAD1 and GAD2 receptors, forming axosomatic and axo-axonic inhibitory synapses, accordingly (Fink et al., 2014; Mende et al., 2016). Under physiological conditions, PENK+/GAD2+ neurons (that is, OFF cells) directly communicate with the primary afferent of DRG neurons and tonically inhibit pain responses (Francois et al., 2017), counteracting 5-HTergic-induced pronociception. When under conditions of mechanical pain, PENK/GAD1+ neurons (namely ON cells) synapse onto Penk + dorsal horn interneurons, disinhibit PENK + neurons, and facilitate the transmission of mechanical pain stimuli. Furthermore, GABAergic PENK + neurons contribute to stress-induced modulation of pain, since acute stress increases activities of GABAergic PENK + neurons, exerting analgesia while chronic stress decreases the expression of GABAergic PENK + neurons and induces hyperalgesia (Francois et al., 2017).

**FIGURE 3:**
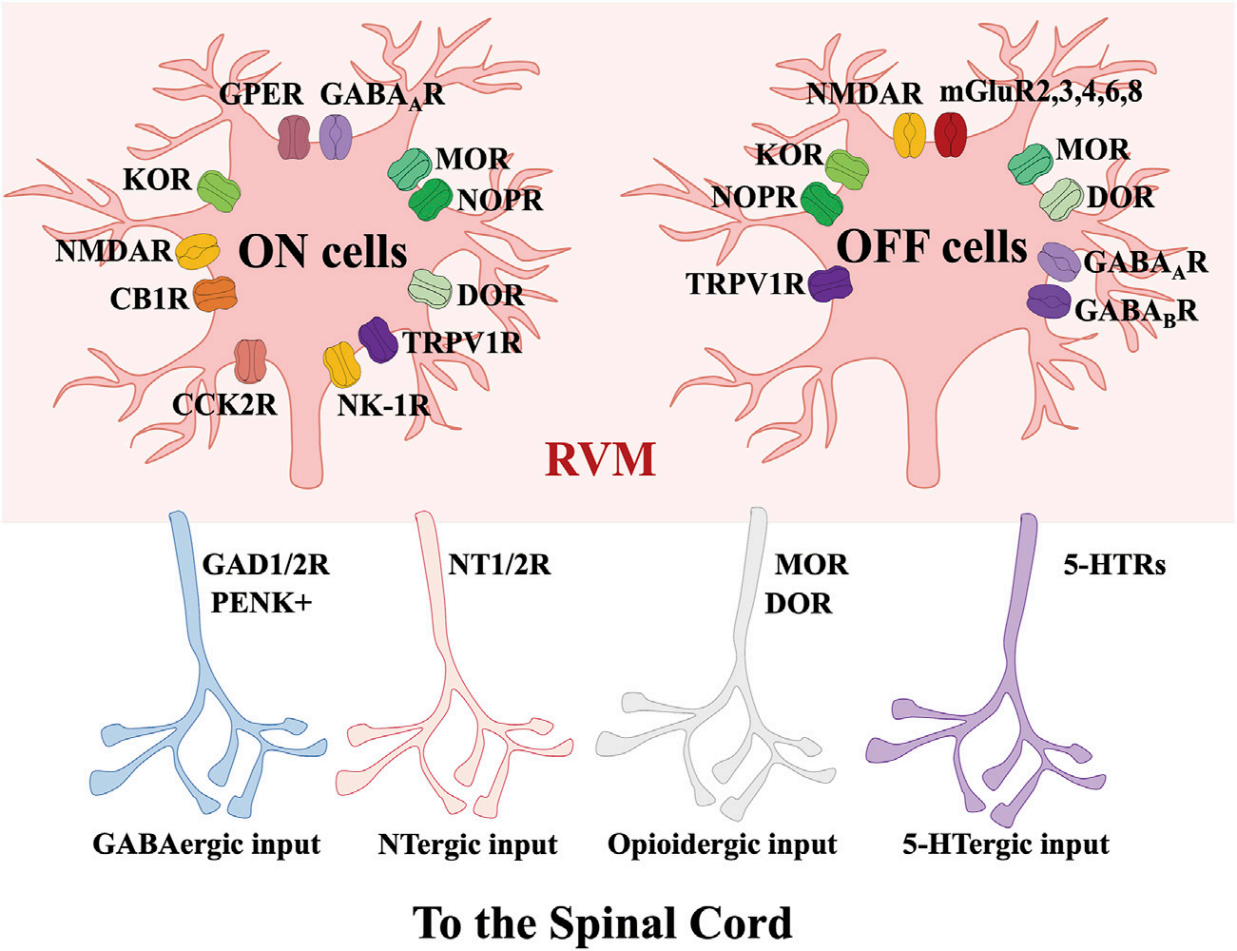
Molecular characteristics of RVM-spinal cord circuits modulating nociception. Via acting on GAD, 5-HTRs, NT and opioid receptors, the projections from RVM ON and OFF cells to the spinal cord contain GABAergic, NTergic, opioidergic and 5-HTergic inputs.

With regard to 5-HTergic neurons, its effects on RVM-spinal cord pathway are confirmed, however the concrete influence on RVM ON, OFF or Neutral cells remain controversial, which were discussed aftermentioned. 5-HT is produced primarily in the NRM, although retrograde labeling studies have demonstrated that 5-HTergic projections to the spinal dorsal horn simply arise from the NRM and terminate densely in the superficial laminae (laminae I and II) and in the deeper laminae (laminae IV-VI) of the dorsal horn. Meanwhile, stimulation of RVM leads to increased 5-HT release in the spinal cord, contributing to bidirectional effects on nociceptive modulation (Ossipov et al., 2010; Kort et al., 2022). In reality, there are two populations of 5-HTergic neurons in the RVM, those involved in spinal projections and in local modulation. Although some RVM 5-HTergic neurons express MOR (13.8% ± 3.9%) and GABA (8%), only about half of these project to the spinal cord, which suggests the existence of 5-HTergic neurons for local modulation (Wei et al., 2010; Lau et al., 2020). With regard to local 5-HTergic neurons in the RVM, direct microinjection of 5-HT increased the release of 5-HT in the NRM, modulating ON and OFF cells in RVM through 5-HT1R and 5-HT2R, which ultimately decrease tail flick latency, and exert an inhibitory influence on pain modulation (Brito et al., 2017; Wang et al., 2017). These findings indicate that 5-HTergic neurons could modulate the pain response by affecting the excitability of ON and OFF cells. For the spinal projection of 5-HT-ergic neurons, Sagalajev et al. (2017) explored some interesting findings in a pharmacological study involving the microinjection of NT in the RVM, which resulted in reduced ON cell discharge and facilitated OFF cell activation, thus inducing antinociceptive effects (Buhler et al., 2005). Meanwhile, injection of EM-2 into the RVM activated MOR in spinal projection of 5-HTergic neurons and increased descending 5-HTergic facilitatory influences, although these descending 5-HTergic projections were neither necessary nor sufficient for RVM MOR- or KOR-mediated modulation of descending pain in acute pain conditions (Gu and Wessendorf, 2007; Chen and Heinricher, 2019a; Kohn et al., 2020). Hence, it is reasonable that under physiological conditions, activation of RVM 5-HTergic neurons will inhibit spinal nociceptive neurotransmission. In experimental models of diabetic neuropathy and chemotherapy-induced neuropathy, the excitability of 5-HTergic neurons in RVM increased, inducing increased recruitment of descending 5-HTergic projections to the spinal cord and resulting in pain hypersensitivity (Sikandar et al., 2012). Through molecular engineering approaches using shRNA plasmids and electroporation, RVM 5-HTergic neurons were found to function as modulators of pain in the promotion and maintenance, rather than triggering, of inflammatory or neuropathic pain (Wei et al., 2010). Taken together, the existing evidence suggests that two types of RVM 5-HTergic neurons can modulate spinal pain transmission in direct and indirect ways, which may be responsible for the bidirectional effects on modulating pain in physiology and pathophysiology conditions. Conversely, bidirectional projections in nociception also closely depend on the activation of various subtypes of the 5-HT receptor (5-HT1R-7R). Generally, 5-HT1AR, 5-HT2AR, and 5-HT4R generate bidirectional effects, while 5-HT1B/DR and 5-HT2CR are primarily antinociceptive (Bardoni, 2019; Heijmans et al., 2021; Fauss et al., 2022). In contrast, 5-HT2BR is activated by facilitating CCK2R in RVM ON cells and induces hyperalgesia (Jiang et al., 2019). Furthermore, the function of 5-HT3R and 5-HT7R remains uncertain. In neuropathic or inflammatory pain states, blocking the 5-HT3R specifically attenuates 5-HT pronociceptive actions in the RVM while activation of the 5-HT3R plays a facilitatory role in nociceptive responses. These findings were in parallel with the observations that a descending release of 5-HT from the RVM combined with the upregulation of 5-HT3R expression in spinal cord neurons was detected after inflammation and nerve injury (Wei et al., 2010; Wang et al., 2019; Liu et al., 2020). Thus, it is confirmed that spinal 5-HT3R mainly mediates descending facilitation under abnormal pain conditions. Specifically, activation of the p38 mitogen-induced protein kinase (p38MAPK) pathway in the RVM increases the expression of tryptophan hydroxylase (Tph), resulting in increased activities of ON cells and expression of 5-HT along with the silencing of OFF cells, which then act on the spinal 5-HT3R to activate ATP-gated P2X7 receptors in the microglia, and ultimately exerting pain hypersensitivity (Liu et al., 2020). As 5-HT3R is also expressed on inhibitory GABAergic interneurons, antinociceptive effects are confirmed to correspond to activation of OFF cells in subcutaneously excited RVM 5-HT3R and GABA release in the GABA spinal cord in acute pain models (Nasirinezhad et al., 2016; Kort et al., 2022). With regard to 5-HT7R, under conditions of neuropathic pain, the study conducted by Brenchat et al. (2010) showed that the spinal 5-HT7R was expressed mainly on the GABAergic and Penk + interneurons, which stimulate OFF cells, release 5-HT, GABA, and enkephalins, and therefore enhance nociceptive inhibition, in contrast to the pronociceptive effects exerted in the periphery. In healthy conditions, spinal 5-HT7R only shows antinociceptive effects, in which the exact mechanism remains to be defined (Wang et al., 2015) (Figure 3).

## 5 Conclusion

As the final relay nuclei of the descending pain modulation system, the RVM converges on the comprehensive modulation input from different nuclei like the PAG, amygdala, PB, LC, and the hypothalamus, as reviewed herein, and then integrates this information to influence the excitability status of ON, OFF, and even Neutral cells in this area, whose descending projections lead to the spinal cord, where they exert facilitation or inhibition of spinal pain transmission, and finally control the nociceptive intensity received by the brain from the periphery. In this process, GABA, 5-HT, endogenous opioids, endogenous cannabinoids, and their corresponding receptors are the main mediators involved in the facilitation and inhibition of pain. Moreover, it has been generally accepted that RVM related circuits and neurotransmitters play a pivotal role in the maintenance and persistence of chronic pain; thus, the RVM is a major therapeutic nucleus for the development of novel strategies for alleviation of pain. Conversely, in order to successfully obtain further scientific understanding of RVM or clinical pain treatment targeting RVM, we first need to identify a suitable model for specific modulation of ON and OFF cells, as well as clarify the molecular and circuit changes associated with the RVM under pathological conditions.
